# Auxiliary self-supervision to metric learning for music similarity-based retrieval and auto-tagging

**DOI:** 10.1371/journal.pone.0294643

**Published:** 2023-11-30

**Authors:** Taketo Akama, Hiroaki Kitano, Katsuhiro Takematsu, Yasushi Miyajima, Natalia Polouliakh

**Affiliations:** 1 Sony Computer Science Laboratories, Inc, Tokyo, Japan; 2 Koozyt, Inc, Tokyo, Japan; Seoul National University of Science & Technology, KOREA, REPUBLIC OF

## Abstract

In the realm of music information retrieval, similarity-based retrieval and auto-tagging serve as essential components. Similarity-based retrieval involves automatically analyzing a music track and fetching analogous tracks from a database. Auto-tagging, on the other hand, assesses a music track to deduce associated tags, such as genre and mood. Given the limitations and non-scalability of human supervision signals, it becomes crucial for models to learn from alternative sources to enhance their performance. Contrastive learning-based self-supervised learning, which exclusively relies on learning signals derived from music audio data, has demonstrated its efficacy in the context of auto-tagging. In this work, we propose a model that builds on the self-supervised learning approach to address the similarity-based retrieval challenge by introducing our method of metric learning with a self-supervised auxiliary loss. Furthermore, diverging from conventional self-supervised learning methodologies, we discovered the advantages of concurrently training the model with both self-supervision and supervision signals, without freezing pre-trained models. We also found that refraining from employing augmentation during the fine-tuning phase yields better results. Our experimental results confirm that the proposed methodology enhances retrieval and tagging performance metrics in two distinct scenarios: one where human-annotated tags are consistently available for all music tracks, and another where such tags are accessible only for a subset of music tracks.

## 1 Introduction

Just as web search engines, article curation, and recommendations have revolutionized the way we gather information, in the field of music as well, search engines, curation, and recommendations are becoming increasingly important in how we listen to music and how creators produce content.

With the advent of music streaming services, we have entered an era where, depending on how we search, we can listen to various music tailored to our contexts. We have begun to consume and produce large amounts of video content on social media and video streaming services. With the widespread use of smartphones, we casually capture daily memories in videos and edit them, leading to an explosive increase in video consumption and production in recent years. Music or background music (BGM) is effectively and skillfully used in these videos [1], deeply influencing our emotions, often without us being consciously aware. There is a demand to add music to such videos, irrespective of whether the creator is professional or amateur. Furthermore, with AI music generation, we are entering an era where music is semi-automatically produced [2, 3], indicating a forthcoming deluge of music. Now, more than ever, there’s a growing need for information organization techniques to deliver the desired music to consumers and creators.

At the core of this information organization technology lie auto-tagging and similarity-based retrieval. Auto-tagging is a task where, upon inputting a music track into the system, it automatically analyzes the track and outputs tag information related to genre, mood, instruments, etc. This serves as the foundation for various music delivery applications such as recommendation, curation, playlist generation, and user behavior analysis [4]. Similarity-based retrieval, on the other hand, is a task where, upon inputting a music track into the system, it automatically analyzes the track, retrieves similar music tracks from the database, and ranks them in order of similarity. Besides forming the basis for music delivery applications like recommendation, query-by-example, and playlist generation [5], similarity-based retrieval itself also becomes a significant application.

To effectively handle the immense volume of available music information, enhancing foundational technologies such as auto-tagging and similarity-based retrieval is essential. However, the frequent absence of consistent and informative tag data for music tracks complicates the training of models for these tasks. Manual tagging has its limitations, from inconsistencies among annotators to challenges in adapting to new genres and variations in tag notation. Further, for non-mainstream genres and music catalogs geared more towards business rather than direct consumers, relying on public tagging is not only challenging but often impractical or impossible [6]. Data from user activity on popular music streaming services offers insights into user preferences, but it comes with issues. Obtaining objective data about a music track’s genre, mood, and other attributes is tough. Additionally, this data is inaccessible unless developers have access to a popular service, new tracks lack feedback, and feedback primarily focuses on popular tracks [7]. Given this context, there’s a demand for technologies that can fill in the gaps of objective music content information. This paper introduces a technology capable of automatically supplementing such music content information, enhancing similarity-based retrieval and auto-tagging performance.

Conventional methods for similarity-based music retrieval largely depend on supervised learning, utilizing learning signals derived from human-annotated tags [8]. In contrast, self-supervised learning gleans its learning signals from inherent properties of the music tracks themselves, thus autonomously augmenting music content information without the need for attached annotations or metadata. Among these self-supervised approaches, contrastive learning has shown promise and has been applied to auto-tagging [9]. In this work, we present a model that integrates metric learning and contrastive-based self-supervised learning. We demonstrate that contrastive-based self-supervised learning is advantageous not only for auto-tagging but also for the similarity-based retrieval task. Furthermore, we introduce refined techniques to improve conventional self-supervised learning methods.

What is an intuitive explanation for our self-supervised signals? The similarity between music tracks is typically defined by their global similarity, which considers how closely related their global attributes are [8]. Auto-tagging performance is assessed based on the ability to infer global tags from each music track [8, 10, 11]. Our neural network model aims to extract such global attribute features without relying solely on manually annotated tags. We formulate learning signals under the assumption that excerpts from the same music track are more likely to possess similar global attribute features compared to excerpts from different music tracks. Additionally, we assume that the global attribute features of a track remain relatively unchanged even after applying augmentation transformations, such as reverberation, band-pass filtering, and pitch shifting. Given that the learning signal is derived from annotations inherent to the music audio (i.e., self-supervised) rather than from human-provided annotations (i.e., human-supervised), this approach is termed self-supervised learning.

To effectively integrate self-supervision signals into our model, a deliberate design consideration is essential. This includes determining where in the architecture to situate embeddings for similarity-based retrieval, given that global attribute features are more directly relevant to these embeddings than classification probabilities. To this end, we have strategically placed embeddings for similarity-based retrieval immediately after the layer where output features are influenced by self-supervised signals. Additionally, we have carefully considered the placement of normalization operations, ensuring that they do not impact the head of the network on the self-supervised loss function side. We have placed them after the branch leading to the head of the network on the supervised loss function side.

Our self-supervised loss diverges from conventional self-supervised losses in several aspects. Self-supervised learning is frequently introduced in the context of representation learning, wherein the acquired representation, or feature, is fixed (the learned neural network is frozen), and the representation is employed for other tasks during the so-called fine-tuning phase [9, 12]. In this paper, we utilize self-supervised learning to enhance task performance and propose adapted learning techniques. Specifically, 1) during the fine-tuning phase, the neural network is not frozen, allowing the entire network to be trained to capitalize on its expressivity. 2) Self-supervised learning signals are employed even in the fine-tuning phase. 3) Augmentation is omitted for self-supervised learning during the fine-tuning phase, enabling our neural network model to be trained with higher quality data. Overall, we consider the self-supervised signal as an auxiliary loss in relation to the primary metric learning loss, which improves performance compared to employing the standard self-supervised approach, where the learned neural network is frozen during the fine-tuning phase.

To further leverage the self-supervised signals, especially to address situations where real-world data doesn’t always have clean and informative tags, we empirically demonstrate that our method is also effective in addressing semi-supervised scenarios where obtaining human-annotated tags for music tracks is expensive and tags may not always be available for all music tracks used in training models. Notably, the improvement over existing methods was even more significant in situations where only 1% of the songs in the database were tagged.

Our primary contributions can be summarized as follows:

- We propose a model architecture and a training algorithm that employ self-supervised learning to boost the performance of similarity-based music retrieval in both supervised and semi-supervised contexts.
- We introduce a self-supervised auxiliary loss for similarity-based music retrieval and music auto-tagging, which serves to augment the outcomes in comparison to the conventional self-supervised approach within the supervised scenario.

The remainder of this paper is organized as follows: Section 2 delves into the literature review, offering insights into prior research and identifying gaps in the current knowledge, complementing this section of introduction. Section 3 describes some preliminary technical terms which serve as the basic knowledge to understand the methodology of the paper. Section 4 presents the methodology, introducing our problem setting and detailing the architecture and objective functions of our proposed model. Section 5 offers experimental setups, providing information on datasets we use for experiments, detailed model configurations, evaluation metrics, and baseline methods. Section 6 describes the experimental results of our proposed model, comparing with baseline methods and variations of our models. Finally, Section 7 concludes the paper, summarizing the main points.

## 2 Related work

Spijkervet and Burgoyne demonstrated the effectiveness of SimCLR-based self-supervised learning for music auto-tagging [9]. We show that self-supervised learning is effective not only for auto-tagging but also for similarity-based music retrieval. Furthermore, our aim is to improve practical performance rather than merely evaluating representation quality. To this end, we propose a self-supervised auxiliary loss accompanied by a simple modified procedure that outperforms their self-supervised approach.

Thomé et al. introduced four triplet learning terms for learning music similarity, which include transformed excerpts, excerpts from the same track, and genre and mood membership [13]. In contrast, our model employs SimCLR-based contrastive learning for self-supervised learning, manages general multi-tag settings through classification-based metric learning, and addresses the auto-tagging task. Our focus is to show the effectiveness of the loss without using tag information and demonstrate effectiveness in semi-supervised settings, which is distinct from them.

Manocha et al. proposed a differentiable speech similarity model with application to improving speech synthesis and enhancement models. They utilized SimCLR for pre-training the body of the model, trained head of the model on JND data (speech similarity dataset), and employed triplet comparison for fine-tuning the model [14]. Their model is designed mainly for the loss in speech synthesis and enhancement models, but our model is designed for auto-tagging and similarity-based retrieval. Their method focuses on speech similarity using carefully designed speech domain datasets, differing from our approach that targets global audio similarity in the music domain by leveraging widely available tag annotations.

To improve retrieval of image using unlabeled image datasets, Duan et al. introduced a self-training framework for metric learning [15]. They used self-supervised learning to train a teacher network. Subsequently, they used the teacher network to generate pseudo labels, which were then utilized for metric learning with ranking loss. Our method applies self-supervision directly to the “student” network eliminating the need for a teacher network. Additionally, their method is designed for the image domain rather than music.

Fu et al. proposed deep metric learning with self-supervised ranking to improve retrieval and ranking of image [16]. They introduced an intra-class ranking loss in a self-supervised manner, in addition to metric learning for handling inter-class variance. However, their self-supervision employs intra-class ranking loss, which is distinct from our contrastive self-supervised loss, and their method is tailored to the image domain rather than music.

In summary, our work is distinct in that we investigate how to design architectures and losses when combining supervised metric learning and classification with cutting edge contrastive based self-supervised learning.

## 3 Preliminary

In this section, we review some basic mathematical operations used in the next section.

### 3.1 Layer normalization

One way to stabilize training and reduce the training time of deep neural networks is to normalize the activities of the neurons. Layer normalization (LayerNorm) is one of the most well-known normalization techniques [17]. Formally, LayerNorm without affine parameters is defined for a vector **x** = [*x*_1_, *x*_2_, …, *x*_*n*_] by

$$\begin{array}{c} \text{LN}\left(\text{x}\right)=\frac{\text{x}-E[\text{x}]}{\sqrt{\text{Var}[\text{x}]}}, \end{array}$$ (1)

where E[**x**] and Var[**x**] denote the mean and variance of **x** over its dimension. LayerNorm without affine parameters was shown to be effective in classification-based metric learning by helping the network better initialize new parameters and reach better optima [18]. In this paper, LayerNorm is used in Eqs (7) and (16).

### 3.2 *ℓ*^2^-norm

*ℓ*^2^-norm is a vector norm defined for a vector **x** = [*x*_1_, *x*_2_, …, *x*_*n*_] by

$$\begin{array}{c} {\left\|\text{x}\right\|}_{2}=\sqrt{\sum_{k=1}^{n}x_{k}^{2}}. \end{array}$$ (2)

In this paper, *ℓ*^2^-norm is used for defining the distance in the embedding space of similarity in Eqs (7) and (16), following the distance definition in Eq (4), and defining the cosine similarity in Eq (14).

### 3.3 Sigmoid activation

Sigmoid activation is an activation function defined for a vector **x** = [*x*_1_, *x*_2_, …, *x*_*n*_] by

$$\begin{array}{c} \sigma \left(\text{x}\right)=\frac{1}{1+\text{exp}(-\text{x})}=\left[\frac{1}{1+\text{exp}(-x_{1})},\frac{1}{1+\text{exp}(-x_{2})},\ldots ,\frac{1}{1+\text{exp}(-x_{n})}\right]. \end{array}$$ (3)

Since the range of the sigmoid activation is [0, 1], this activation is used for outputting the probability of binary classes. In Eq (3), when *n* > 1, the activation yields multiple probabilities of binary classes, which are used for multi-tag classification problems. In this paper, the sigmoid activation is used in Eqs (6) and (17).

## 4 Methodology

In this section, we introduce our problem setting and our proposed model, detailing the architecture, objective functions, and algorithms.

### 4.1 Problem setting

Let us consider a dataset

$$D=\{({\text{x}}_{k},{\text{y}}_{k}){\}}_{k=1}^{N_{\text{label}}}\cup \{{\text{x}}_{k}{\}}_{k=1}^{N_{\text{unlabel}}},$$

a set of *N*_label_ pairs of a music track ${\text{x}}_{k}\in X$ and its multi-tag ${\text{y}}_{k}\in Y$ and a set of *N*_unlabel_ music tracks ${\text{x}}_{k}\in X$. Our goal is to learn a similarity function $F_{\text{sim}}:X\to Z$ given D, where $F_{\text{sim}}\left({\text{x}}_{k}\right)\in Z\subset R^{D}$ is an embedding vector with dimensionality *D*, and some distance in the latent space Z captures similarity of data points ${\text{x}}_{k}\in X$. Here, R is the set of all real numbers. *F*_sim_ maps a music track to an embedding vector for the similarity-based retrieval task. Our goal is also to learn a tag function $F_{\text{tag}}:X\to Y$ given D, where $F_{\text{tag}}\left({\text{x}}_{k}\right)\in Y\subset {\left[0,1\right]}^{T}$ is a probability vector of *T* tags whose *t*-th element is the probability that *t*-th tag is assigned to **x**_*k*_. *F*_tab_ maps a music track to a probability vector for the auto-tagging task.

### 4.2 Outline of our model

Instead of learning *F*_sim_ and *F*_tab_ directly, our model learns functions *f*_sim_ and *f*_tag_ whose input is an excerpt ${\text{x}}^{\text{exc}}\in X^{\text{exc}}$, cropped from music tracks, following previous work [8]. *f*_sim_ and *f*_tag_ are the same as *F*_sim_ and *F*_tag_ in that they output a similarity vector and tag probabilities. However, *f*_sim_ and *f*_tag_ differ from *F*_sim_ and *F*_tag_ in that they take as input an excerpt cropped from a music track, rather than the entire music track. We consider a music track **x**_*k*_ as an ensemble of short excerpts derived from it. By feeding each of these excerpts into *f*_sim_ and *f*_tag_ and subsequently aggregating their outputs, we formulate *F*_sim_ and *F*_tag_. Formally, let ${\left({\text{x}}_{k,e}^{\text{exc}}\right)}_{e=1}^{E}$ be a sequence of excerpts cropped from a music track **x**_*k*_, where *E* is the total number of excerpts cropped from the track. Then, our model learns an excerpt similarity function $f_{\text{sim}}:X^{\text{exc}}\to Z$, and we define

$$\begin{array}{c} F_{\text{sim}}\left({\text{x}}_{k}\right)=\frac{\frac{1}{E}\sum_{e=1}^{E}f_{\text{sim}}\left({\text{x}}_{k,e}^{\text{exc}}\right)}{\|\frac{1}{E}\sum_{e=1}^{E}f_{\text{sim}}\left({\text{x}}_{k,e}^{\text{exc}}\right){\|}_{2}}, \end{array}$$ (4)

where ‖⋅‖_2_ is *ℓ*^2^-norm. Similarly, our model also learns an excerpt tag function $f_{\text{tag}}:X^{\text{exc}}\to Y$, and we define

$$\begin{array}{c} F_{\text{tag}}\left({\text{x}}_{k}\right)=\frac{1}{E}\sum_{e=1}^{E}f_{\text{tag}}\left({\text{x}}_{k,e}^{\text{exc}}\right). \end{array}$$ (5)

In experiments, excerpts are non-overlapping sliding windows in each track to avoid higher computational cost and to follow the convention of previous works [8, 9].

**Fig 1:**
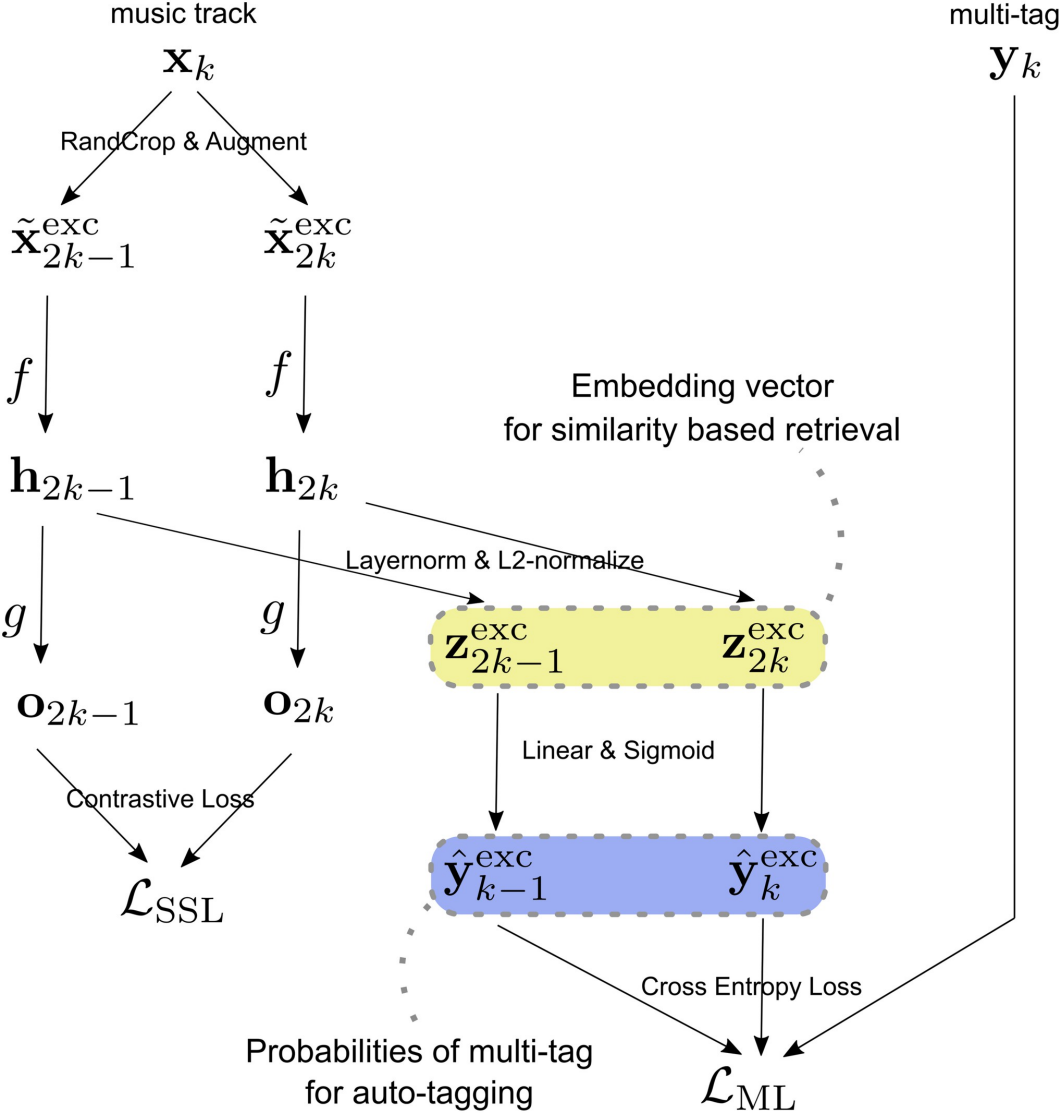
Model overview. For each batch comprising pairs of a music track **x** and its corresponding multi-tag **y**, the music tracks undergo transformations (indicated by arrows) to compute the self-supervised learning loss $L_{\text{SSL}}$ and the metric learning loss $L_{\text{ML}}$. The losses are used to define the overall loss function $L_{\text{SSML}}=\lambda L_{\text{SSL}}+L_{\text{ML}}$ (Eq (20)) to train our proposed model. After training the model, given a music track **x**, the embedding vector **z**^exc^ and the estimated probabilities of multi-tag ${\hat{\text{y}}}^{\text{exc}}$ are used for similarity-based retrieval and auto-tagging, respectively.

Next, we explain the outline of how to model and learn the similarity and tag functions *f*_sim_ and *f*_tag_, which are also visualized in Fig 1. Similarity learning (metric learning) is achieved by tagging (classification) based methodology, as revealed in prior studies [18, 19], where we use the output from the layer just before the final layer of the classification model as an embedding for similarity. Formally, our model learns *f*_tag_ such that

$$\begin{array}{c} f_{\text{tag}}\left({\text{x}}_{k,e}^{\text{exc}}\right)=\sigma \left(Wz_{k,e}^{\text{exc}}\right)\;\text{with}\;{\text{z}}_{k,e}^{\text{exc}}=f_{\text{sim}}\left({\text{x}}_{k,e}^{\text{exc}}\right), \end{array}$$ (6)

where $W\in R^{T\times D}$ is a parameter for mapping the output of *f*_sim_ to the output of *f*_tag_, *σ* denotes the sigmoid activation, and ${\text{z}}_{k,e}^{\text{exc}}\in R^{D}$ is an embedding vector for similarity-based retrieval. Model architectures for similarity-based retrieval and auto-tagging are mostly shared in this formulation, so it is advantageous in practice in terms of time, memory, and storage in training and inference phases, particularly when using functionalities of both similarity-based retrieval and auto-tagging. In Sections 4.3 and 4.4, we explain how to train *f*_sim_ and *W* (thus *f*_tag_) in detail, where *f*_sim_ is defined as a function *f*, followed by layer normalization [17], followed by normalizing with *ℓ*^2^-norm. Formally,

$$\begin{array}{c} f_{\text{sim}}\left(\cdot \right)=\frac{\text{LN}(f(\cdot ))}{{\left\|\text{LN}(f(\cdot ))\right\|}_{2}}, \end{array}$$ (7)

where LN denotes layer normalization. Here, both *f* and *f*(⋅) refer to the same function, and similarly, both *f*_sim_ and $f_{\text{sim}}\left(\cdot \right)$ refer to the same function. Then our goal in the Sections 4.3 and 4.4 boils down to learning *f* and *W*, where we choose to use the SampleCNN architecture for *f* [20]. *f* is trained using a self-supervised learning loss and a metric learning loss (a loss function based on metric learning approach) whereas *W* is trained only using a metric learning loss. Since inner product is the distance metric between each row of *W* and ${\text{z}}_{k,e}^{\text{exc}}$ in Eq (6), we use inner product as the distance metric in the similarity space when conducting similarity-based retrieval.

### 4.3 Self-supervised learning

Consider a mini-batch ${\left\{{\text{x}}_{k}\right\}}_{k=1}^{B}$ from the dataset D, where *B* is the batch size, and a set of augmentation operations A (See Section 5.2 for the choice of A in experiments). We follow the Contrastive Learning of Musical Representation (CLMR) [9], which uses the simple framework for contrastive learning of visual representations (SimCLR) for self-supervised learning [12]. For each sample **x**_*k*_ in a mini-batch, we randomly crop two excerpts from **x**_*k*_ (where the random crop refers to cropping an excerpt from a music track, where the excerpt position in a music track is drawn uniformly from all possible positions), apply an augmentation operation to each of the excerpts (where the augmentation operations *a* and *a*′ are sampled uniformly from A, i.e., $a,a^{\prime }∼A$), and then feed each into the function *f* followed by another function *g*. Formally we compute the following transformations:

$${\tilde{\text{x}}}_{2k-1}^{\text{exc}}=a(\text{RandCrop}({\text{x}}_{k})),$$ (8)

$${\text{h}}_{2k-1}=f({\tilde{\text{x}}}_{2k-1}^{\text{exc}}),$$ (9)

$${\text{o}}_{2k-1}=g({\text{h}}_{2k-1}),$$ (10)

$${\tilde{\text{x}}}_{2k}^{\text{exc}}=a^{\prime }(\text{RandCrop}({\text{x}}_{k})),$$ (11)

$${\text{h}}_{2k}=f({\tilde{\text{x}}}_{2k}^{\text{exc}}),$$ (12)

$${\text{o}}_{2k}=g({\text{h}}_{2k}),$$ (13)

where a pair $\left({\tilde{\text{x}}}_{2k-1}^{\text{exc}},{\tilde{\text{x}}}_{2k}^{\text{exc}}\right)$ is referred to as a *positive pair*. The random crop (denoted as RandCrop(⋅)) and augmentation operations are assumed to preserve the global attributes. For the architecture of *g*, we use a linear layer followed by a ReLU layer followed by a linear layer, where no bias term is used in the linear layers.

Given a set $\{{\tilde{\text{x}}}_{l}^{\text{exc}}{\}}_{l=1}^{2B}$ including a positive pair of examples ${\tilde{\text{x}}}_{i}^{\text{exc}}$ and ${\tilde{\text{x}}}_{j}^{\text{exc}}$, the contrastive prediction task aims to identify ${\tilde{\text{x}}}_{j}^{\text{exc}}$ in $\{{\tilde{\text{x}}}_{l}^{\text{exc}}{\}}_{l\neq i}$ for a given ${\tilde{\text{x}}}_{i}^{\text{exc}}$. Formally, letting sim(**u**, **v**) = **u**^⊤^**v**/‖**u**‖_2_‖**v**‖_2_, ${\text{o}}_{i}=g(f({\tilde{\text{x}}}_{i}^{\text{exc}}))$, ${\text{o}}_{j}=g(f({\tilde{\text{x}}}_{j}^{\text{exc}}))$, and ${\text{o}}_{l}=g(f({\tilde{\text{x}}}_{l}^{\text{exc}}))$, a contrastive loss function *L*_SSL_(*i*, *j*) can be defined for a contrastive prediction task as

$$\begin{array}{c} L_{\text{SSL}}\left(i,j\right)=-\text{log}\frac{\text{exp}(\text{sim}({\text{o}}_{i},{\text{o}}_{j})/\tau )}{\sum_{l=1}^{2B}1_{\left[l\neq i\right]}\text{exp}(\text{sim}({\text{o}}_{i},{\text{o}}_{l})/\tau )}, \end{array}$$ (14)

where *τ* is a temperature parameter set to the default value proposed in SimCLR [12]. *L*_SSL_(*i*, *j*) is computed for all augmented pairs, i.e., $\left(i,j\right)\in {\left\{\left(2k-1,2k\right)\right\}}_{k=1}^{B}⋃{\left\{\left(2k,2k-1\right)\right\}}_{k=1}^{B}$ and averaged, yielding the overall loss function

$$\begin{array}{c} L_{\text{SSL}}=\frac{1}{2B}\sum_{k=1}^{B}\left[L_{\text{SSL}}\left(2k-1,2k\right)+L_{\text{SSL}}\left(2k,2k-1\right)\right]. \end{array}$$ (15)

### 4.4 Metric learning with self-supervised auxiliary loss

We propose to combine classification-based metric learning with self-supervised learning. Layer normalization (denoted by LN(·)) is applied to **h**_*i*_, followed by normalization with *ℓ*^2^-norm to yield an embedding vector ${\text{z}}_{i}^{\text{exc}}\in R^{D}$ for similarity-based retrieval. Formally,

$$\begin{array}{c} {\text{z}}_{i}^{\text{exc}}=\frac{\text{LN}({\text{h}}_{i})}{{\left\|\text{LN}({\text{h}}_{i})\right\|}_{2}}. \end{array}$$ (16)

${\text{z}}_{i}^{\text{exc}}$ is then multiplied by *W*, followed by element-wise sigmoid activation to produce classification output ${\hat{\text{y}}}_{i}^{\text{exc}}$, i.e.,

$$\begin{array}{c} {\hat{\text{y}}}_{i}^{\text{exc}}=\sigma (W{\text{z}}_{i}^{\text{exc}}). \end{array}$$ (17)

We use binary cross entropy loss for each tag and average them to compute *L*_ML_(*i*):

$$\begin{array}{c} L_{\text{ML}}\left(i\right)=\frac{1}{T}\sum_{t}^{T}[-{\text{y}}_{i}\left[t\right]\;\text{log}\;({\hat{\text{y}}}_{i}^{\text{exc}}\left[t\right])-(1-{\text{y}}_{i}\left[t\right])\;\text{log}\;(1-{\hat{\text{y}}}_{i}^{\text{exc}}\left[t\right])]. \end{array}$$ (18)

Let $K_{\text{label}}$ be an index set such that $\left\{{\text{x}}_{k}:k\in K_{\text{label}}\subseteq \left\{1,2,\ldots ,B\right\}\right\}$ is the set of all the labeled samples in $\{{\text{x}}_{k}{\}}_{k=1}^{B}$. $L_{\text{ML}}\left(i\right)$ is computed for the samples in the labeled subset and averaged, yielding the loss function

$$\begin{array}{c} L_{\text{ML}}=\frac{1}{\left|K_{\text{label}}\right|}\sum_{k\in K_{\text{label}}}\left[L_{\text{ML}}\left(2k-1\right)+L_{\text{ML}}\left(2k\right)\right]. \end{array}$$ (19)

Finally, the loss function for our proposed model is a combination of the self-supervised loss $L_{\text{SSL}}$ and the metric learning loss $L_{\text{ML}}$, which is defined as:

$$\begin{array}{c} L_{\text{SSML}}=\lambda L_{\text{SSL}}+L_{\text{ML}}. \end{array}$$ (20)

Here λ∈R is a balancing factor between two losses $L_{\text{SSL}}$ and $L_{\text{ML}}$.

In practice, the self-supervised learning needs a longer training time, so we first train our model with $L_{\text{SSL}}$ only, whose phase is referred to as pre-training phase. We then train with $L_{\text{SSML}}$, whose phase is referred to as fine-tuning phase.

## 5 Experimental setup

In this section, we offer experimental setups, providing information on datasets we use for experiments, detailed model configurations, evaluation metrics, and baseline methods.

### 5.1 Dataset

In experiments, we employ two commonly used datasets for music retrieval: the MagnaTagATune dataset [6] and the MTG-Jamendo dataset [21].

#### 5.1.1 MagnaTagATune dataset

The MagnaTagATune dataset consists of 25,000 music tracks from 6,622 unique songs [6]. We use top 50 tags and the same train/validation/test split as in previous work [9], and the train/validation/test datasets are used for both of the similarity-based retrieval and auto-tagging. Utilizing the conventional train/validation/test data splits is essential to maintain fair comparisons with prior studies. To explore the composition of these splits, we looked into the metadata of the datasets to identify common artists within them. It appears that there are 48 common artists, with the train and validation sets containing 203 unique artists, and the test set including 75 unique artists. We obtained the MagnaTagATune dataset using the code in the CLMR repository https://github.com/Spijkervet/CLMR/blob/master/clmr/datasets/magnatagatune.py, where the dataset itself is downloaded from the sota-music-tagging-models repository https://github.com/minzwon/sota-music-tagging-models/tree/master/split/mtat.

#### 5.1.2 MTG-Jamendo dataset

MTG-Jamendo contains 55,000 full music tracks (320kbps, MP3) with 195 tags covering genre, instrument, and mood/theme [21]. We use the pre-defined train/validation/test splits and the top 50 tags. The train/validation/test data splits are used for both of the similarity-based retrieval and auto-tagging. Employing the conventional train/validation/test data splits is essential to ensure fair comparisons with prior works. In order to examine the characteristics of these splits, we looked into the metadata of the datasets to identify common artists within them. It appears that there are no common artists, with the train and validation sets containing 2815 unique artists, and the test set including 702 unique artists. We obtained the MTG-Jamendo dataset from the mtg-jamendo-dataset reposotory https://github.com/MTG/mtg-jamendo-dataset.

### 5.2 Model configurations

The set of augmentation operations A follows CLMR [9] for fair comparison. Specifically, the following operations are applied sequentially with probability *p* to create an element of A:

- polarity inversion (*p* = 0.8)
- additive Gaussian noise with decibel sampled uniformly from [80, 40] (*p* = 0.01)
- gain with decibel sampled uniformly from [−6, 0] (*p* = 0.3)
- low pass filtering or high pass filtering chosen with the same probability, where their cut-off frequency is sampled uniformly from [2200, 4000] Hz and [200, 1200] Hz, respectively (*p* = 0.8)
- delayed signal added to the original signal with a volume factor of 0.5 in which the delay time is randomly sampled from {200, 250, 300, …, 500} ms (*p* = 0.3)
- pitch shifting with shifting semitones sampled uniformly from [−7, 7] (*p* = 0.6)
- reverb with the impulse response’s room size, reverberation, and damping factor sampled uniformly from [0, 100] (*p* = 0.6)

We set the excerpt length to 59049, audio to monaural, and audio sampling rate to 22.05 kHz following CLMR [9] for fair comparison. We set the dimensionality *D* of the embedding vector for the similarity-based retrieval to 512 and set the number of tags *T* to 50.

To determine the value of *λ* in Eq (20), we first introduce the base balancing factor *r* of the two terms $L_{\text{ML}}$ and $L_{\text{SSL}}$. *r* is defined to be $r=L_{\text{ML}}^{\text{only}}/L_{\text{SSL}}^{\text{only}}$, where $L_{\text{ML}}^{\text{only}}$ and $L_{\text{SSL}}^{\text{only}}$ are the converged loss values when the model is trained using either $L_{\text{ML}}$ or $L_{\text{SSL}}$, respectively, and all available labels are used when trained with $L_{\text{ML}}$. The values of *r* were 22.00 for MagnaTagATune dataset and 18.95 for MTG-Jamendo dataset. Then, the candidates for λ in Eq (20) were set to {*α*/*r*: *α* ∈ {0.05, 0.1, 1, 10}}. For conciseness, {*α*/*r*: *α* ∈ {0.1, 1, 10}} for the MagnaTagATune dataset and {*α*/*r*: *α* ∈ {0.05, 0.1, 1}} for the MTG-Jamendo dataset are shown in Tables 1 and 2, respectively.

**Table 1:** Results for supervised scenario of MagnaTagATune dataset.

Models	Techniques			Similarity-based retrieval								Auto-tagging	
	a	c	p	R@1	R@2	R@4	R@8	M@1	M@4	M@7	M@10	ROC	PR
inception				51.7	66.3	78.3	87.5	38.3	29.9	27.1	25.6	0.905	0.375
CLMR			✓									0.894	0.368
our A				52.1	66.4	78.7	87.6	39.1	30.4	27.5	25.8	0.901	0.371
our B	✓			51.0	66.1	78.8	87.8	38.0	29.9	27.0	25.5	0.900	0.373
our C			✓	52.4	66.8	79.3	**88.6**	39.1	30.6	27.9	26.3	0.904	0.377
our D (*α* = 0.1)	✓	✓	✓	52.2	66.7	78.8	88.2	39.0	30.8	27.9	26.3	0.905	0.381
our E (*α* = 0.1)		✓	✓	**53.0**	67.1	78.8	88.1	**39.9**	30.9	**28.0**	**26.5**	**0.906**	0.381
our F (*α* = 1)	✓	✓	✓	**53.0**	66.7	79.2	88.3	39.6	30.7	27.7	26.1	0.905	0.381
our G (*α* = 1)		✓	✓	**53.0**	**67.5**	**79.4**	88.5	39.8	**31.0**	**28.0**	26.3	**0.906**	**0.382**
our H (*α* = 10)	✓	✓	✓	52.3	66.6	78.5	87.7	39.0	30.0	26.9	25.1	0.891	0.352
our I (*α* = 10)		✓	✓	52.8	66.6	78.6	87.7	39.2	30.1	27.1	25.4	0.897	0.361

Our A, B, …, and I are compared with baseline methods inception and CLMR. Techniques a, c, and p indicate “Fine-tune Augment”, “Fine-tune Contrastive”, and “Load Pre-train”, respectively and they are learning techniques that characterize the variations of especially our proposed methods (See Section 5.5). Our G generally achieves the highest scores for the both tasks.

**Table 2:** Results for supervised scenario of MTG-Jamendo dataset.

Models	Techniques			Similarity-based retrieval								Auto-tagging	
	a	c	p	R@1	R@2	R@4	R@8	M@1	M@4	M@7	M@10	ROC	PR
inception				47.5	61.2	73.5	83.6	36.6	26.4	22.8	20.7	**0.829**	**0.292**
our J (*α* = 0.05)	✓	✓	✓	49.3	62.3	73.7	83.5	38.3	27.7	23.8	21.6	0.825	0.285
our K (*α* = 0.05)		✓	✓	52.1	64.5	75.7	84.6	41.4	30.5	26.4	23.8	0.826	0.286
our L (*α* = 0.1)	✓	✓	✓	49.7	62.5	74.2	83.8	39.0	28.2	24.2	21.9	0.826	0.288
our M (*α* = 0.1)		✓	✓	**52.3**	**65.1**	**76.0**	**84.8**	**42.5**	**31.3**	**26.9**	**24.3**	0.828	0.287
our N (*α* = 1)	✓	✓	✓	47.6	60.2	72.2	82.4	36.3	25.4	21.6	19.4	0.822	0.278
our O (*α* = 1)		✓	✓	50.0	62.2	73.5	82.8	39.1	27.6	23.5	21.2	0.825	0.285

Our J, K, …, and O are compared with baseline methods inception and CLMR. Techniques a, c, and p indicate “Fine-tune Augment”, “Fine-tune Contrastive”, and “Load Pre-train”, respectively and they are learning techniques that characterize the variations of especially our proposed methods (See Section 5.5). Note that our G (in Table 1) and M (in Table 2) use exactly the same methodology (ours with “Fine-tune Contrastive” and “Load Pre-train”) except the value of hyper-parameter *α* and they tend to achieve the highest scores for each dataset.

In our model’s pre-training where only $L_{\text{SSL}}$ is used, the batch size is set to 48, we employ the Adam optimizer with a learning rate of 0.0003 and *β*_1_, *β*_2_ = (0.9, 0.999). The model is trained for 10, 000 and 1, 000 epochs for MagnaTagATune and MTG-Jamendo, respectively.

For our model’s fine-training where the overall loss $L_{\text{SSML}}$ is used, the batch size is set to 48. We use the Adam optimizer with a learning rate of 0.001 and *β*_1_, *β*_2_ = (0.9, 0.999), in which the learning rate is multiplied by 0.1 when the validation loss does not improve for 5 epochs. We use a weight decay with a weight of 1.0 × 10^−6^, and the model is trained for 200 epochs maximum. The training is stopped when the validation loss does not improve for 10 epochs, which is referred to as early stopping.

### 5.3 Evaluation metrics

In this section, we explain our evaluation metrics for the two tasks: similarity-based Retrieval and auto-tagging.

#### 5.3.1 Similarity-based retrieval

To evaluate the similarity-based retrieval, we use the recall@K (R@K) metric to measure retrieval quality following the standard evaluation setting in image retrieval [18, 19] and a similarity-based music retrieval model [8]. This metric is useful for evaluating search methods because it measures the quality of the top K retrieved results, which are more important and more likely to be seen by users than lower ranked retrieved results.

To further assess retrieval quality, we propose using a variant of the MAP@K (Mean Average Precision at K; M@K) metric adapted for similarity-based retrieval with a multi-tag annotated music track dataset. The MAP@K metric has been widely used to evaluate recommender systems [22], and its variant, MAP@R, has been applied to image retrieval [15, 23]. da Silva et al. proposed using this metric for tag-based music retrieval [5]. The calculation of our MAP@K (M@K) is roughly as follows: we compute the tag match rate between the query music track and the retrieved music tracks. We calculate the match rate at rank 1, the cumulative match rate from rank 1 to 2, the cumulative match rate from rank 1 to 3, and so on, up to the cumulative match rate from rank 1 to K. By averaging these match rates, tracks that match tags at higher ranks receive higher scores. Formally, let *N* be the number of music tracks in the test split; our MAP@K (M@K) is defined as:

$$\begin{array}{c} MAP@K=\frac{1}{NTK}\sum_{i=1}^{N}\sum_{t=1}^{T}\sum_{k=1}^{K}P_{i,t}\left(k\right), \end{array}$$ (21)

where *P*_*i*,*t*_(*k*) equals the precision at *k* for the *t*-th tag of the *i*-th music track query if the *k*-th ranked retrieved result is correct and is 0 otherwise. Here, the precision at *k* for the *t*-th tag of the *i*-th music track query is defined as $\frac{c_{k}}{k}$, where *c*_*k*_ is the number of music tracks that have the *t*-th tag among the top *k* retrieved results based on the *i*-th query of a music track with the *t*-th tag.

Compared to recall@K, our MAP@K possesses different properties such as: i) weighting higher ranks of the retrieved results more, and ii) the score is based on tags for individual music tracks rather than the union of tags for multiple tracks. The first property may be preferable as users of similarity-based retrieval systems tend to listen to higher-ranked music tracks. The second property might also be beneficial since the purpose of similarity-based music retrieval is often to find a music track with similar attributes to those of the query music track, rather than finding a set of tracks whose intersection of attributes aligns with those of the query music track.

#### 5.3.2 Auto-tagging

Music auto-tagging has been extensively studied, and diverse model architectures has been developed [8, 10, 11]. We follow the standard benchmarking and evaluation criteria and report average tag-wise Area Under the Receiver Operating Characteristic Curve (ROC-AUC) and Precision Recall Area Under the Curve (PR-AUC) scores to measure tag-based retrieval performance.

### 5.4 Baseline methods

We compare our model with a state-of-the-art model for similarity-based retrieval and auto-tagging [8]. We also compare our model with CLMR [9], a model for auto-tagging which uses SimCLR as self-supervised learning for pre-training [12].

### 5.5 Variations of learning techniques

In this section, we discuss three learning techniques “Fine-tune Augment”, “Fine-tune Contrastive”, and “Load Pre-train” that define the variations of our proposed methods and the baseline approaches.

#### 5.5.1 Fine-tune augment

“Fine-tune augment” involves applying augmentation operations (as detailed in Section 5.2) during the fine-tuning phase. Note that the inception model and CLMR do not utilize this technique.

#### 5.5.2 Fine-tune contrastive

“Fine-tune contrastive” entails conducting contrastive self-supervised learning, where the loss is given by Eq (15), during the fine-tuning phase. It is noteworthy that neither the inception model nor CLMR employ this technique.

#### 5.5.3 Load pre-train

“Load pre-train” refers to loading the pre-trained model’s weights at the beginning of the fine-tuning phase. The pre-training is executed using the contrastive self-supervised loss specified by Eq (15). It is pertinent to mention that while CLMR uses this technique, the inception model does not. Moreover, in our proposed methods, we do not freeze the models, even when the pre-trained weights are loaded.

## 6 Results

In this section, we describe and visualize the experimental results of our proposed model, comparing with baseline methods and variations of our models.

### 6.1 Supervised: Scenario where tags are always available for music tracks

We begin with the supervised scenario, where tags are always available for music tracks. Table 1 shows the results for the supervised scenario of the MagnaTagATune dataset, where techniques a, c, and p indicate “Fine-tune Augment”, “Fine-tune Contrastive”, and “Load Pre-train”, respectively and they are learning techniques that characterize the variations of especially our proposed methods (See Section 5.5). In Table 1, our A, B, …, and I represent variations of our model under different settings. Specifically, they differ in the learning techniques a, c, and p employed and in the value of *α* explained in Section 5.2.

Our G outperformed the previous methods, inception and CLMR, on both similarity-based retrieval and auto-tagging tasks. Our A uses the same learning algorithm as that of inception except for the input representation and network architectures, the results of which suggest that the changes do not always lead to higher performance. Our B, “technique a: Fine-tune Augment” added to our A, slightly improved some metrics and slightly degraded some other metrics, although augmentation is usually an effective strategy. Our C, “technique p: Load Pre-train” added to ours A, improves the performance decently. “technique p: Load Pre-train” is the same strategy as CLMR, but our C outperforms it presumably because ours does not freeze the pre-trained network and takes advantage of the expressivity of the pre-trained network.

In the comparison of the differences in *α* values across our D, E, …, and I, the median value of *α* = 1 (represented by our F and G) exhibited the best performance. We found that conducting self-supervised learning while fine-tuning, corresponding to having “technique c: Fine-tune Contrastive”, boosts the performance as in our F and G, especially when no augmentation is performed while fine-tuning, corresponding to having no “technique a: Fine-tune Augment” as in our G. The observed trend of enhanced performance in the absence of “technique a: Fine-tune Augment” remained consistent across other values of *α*. In similarity-based retrieval, models that perform well on the R@K metric tend to also yield good results on the M@K metric. Our proposed method, G, demonstrates robust performance not only in the benchmark metric R@K but also in the application-oriented metric M@K.

Table 2 shows the results for the supervised scenario of MTG-Jamendo dataset. In Table 2, our J, K, …, and O represent variations of our model under different settings. Specifically, they differ in the learning techniques a, c, and p employed and in the value of *α* explained in Section 5.2.

Our M was the most effective for similarity-based retrieval and had comparable performance to inception in terms of auto-tagging. In the comparison of the differences in *α* values across our J, K, …, and O, the median value of *α* = 0.1 (represented by our L and M) exhibited the best performance. We found that no augmentation is performed while fine-tuning, corresponding to having no “technique a: Fine-tune Augment”, boosts the performance as in our M. The observed trend of enhanced performance in the absence of “technique a: Fine-tune Augment” remained consistent across other values of *α*. In similarity-based retrieval, models that perform well on the R@K metric also yield good results on the M@K metric. Our proposed method, M, demonstrates robust performance not only in the benchmark metric R@K but also in the application-oriented metric M@K.

Note that our G (in Table 1) and M (in Table 2) use exactly the same methodology (ours with “technique c: Fine-tune Contrastive” and “technique p: Load Pre-train”) except the value of hyper-parameter *α* and they tend to achieve the highest scores for each dataset. The result shows that, even with different datasets, there is no need to tune anything other than the hyper-parameter *α*, providing a glimpse of our method’s versatility.

### 6.2 Semi-supervised: Scenario where tags are not always available for music tracks

**Fig 2:**
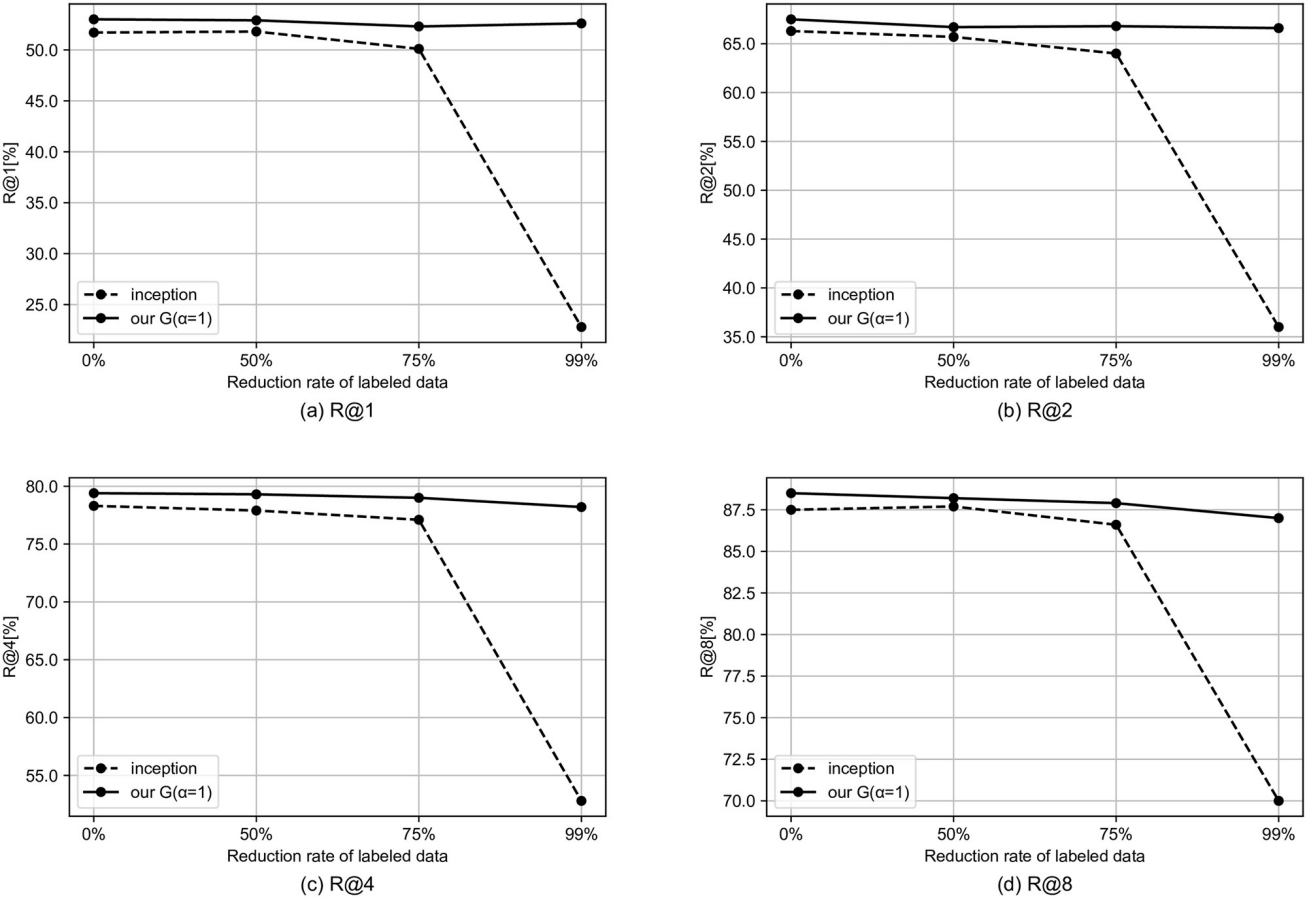
Similarity-based retrieval R@K results for semi-supervised scenario of MagnaTagATune dataset.

**Fig 3:**
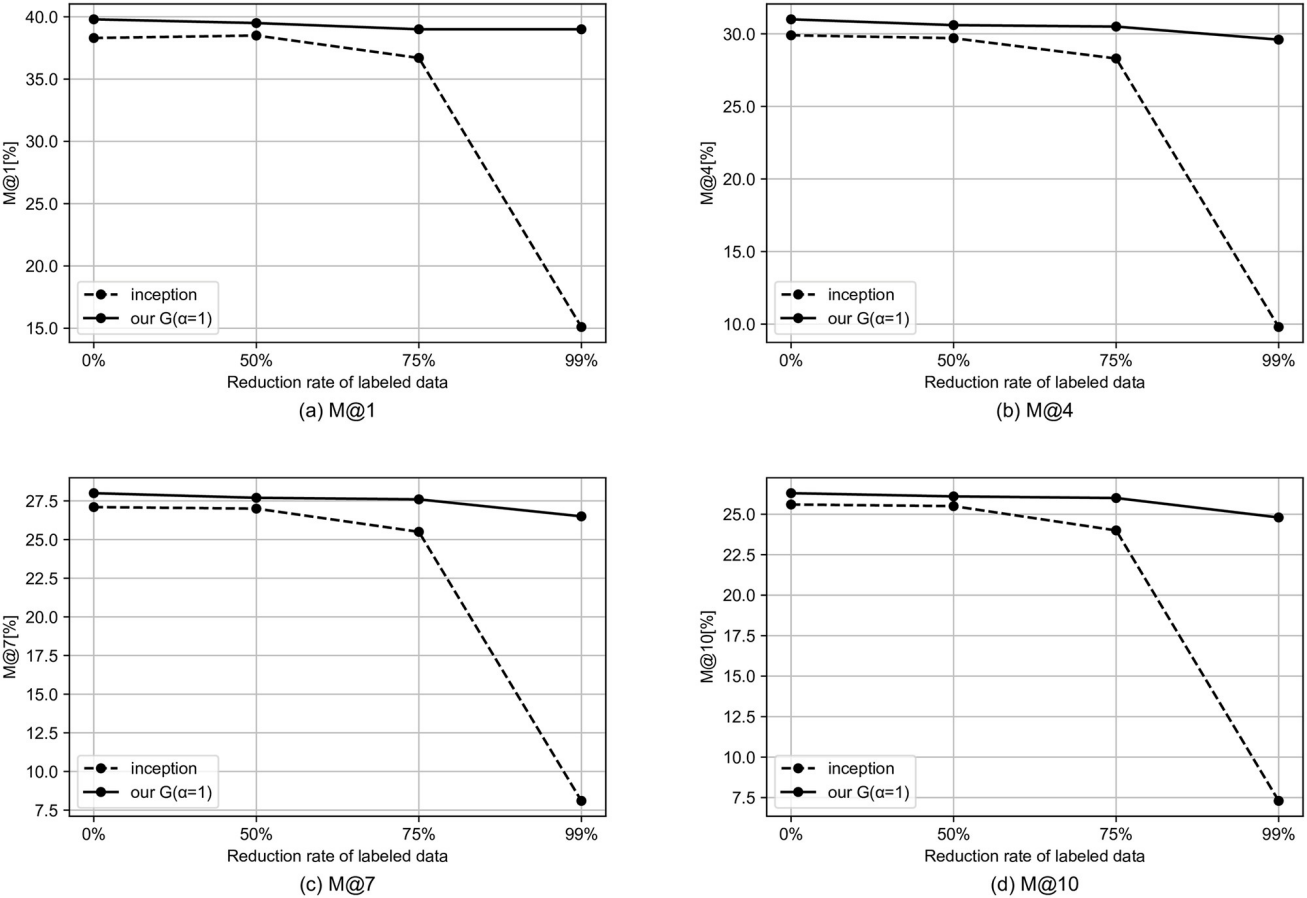
Similarity-based retrieval M@K results for semi-supervised scenario of MagnaTagATune dataset.

**Fig 4:**
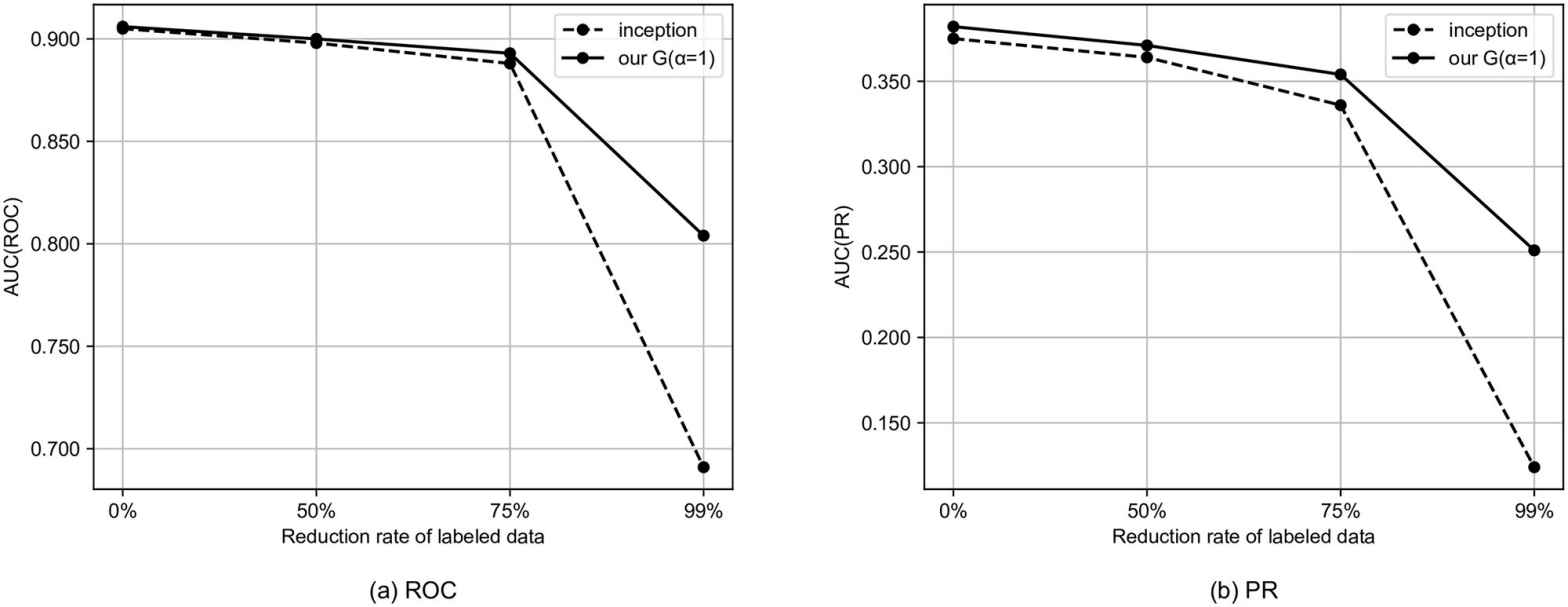
Auto-tagging results for semi-supervised scenario of MagnaTagATune dataset.

We simulate the semi-supervised setting by reducing the rate of tags to be used. In this section, we use the model that performed best in the previous section. Specifically, for the MagnaTagATune dataset and the MTG-Jamendo dataset, we use our G and our M, respectively. Figs 2–4 shows the results for the semi-supervised scenario of the MagnaTagATune dataset. As the amount of labeled data decreases, the performance gap between our model and the baseline tends to widen, and it can be said that our method is more likely to have a larger effect when there is less labeled data. For similarity-based retrieval, the performance of our model only degraded slightly even with a 99% reduction in labeled data (i.e., with only 1% of labeled data).

**Fig 5:**
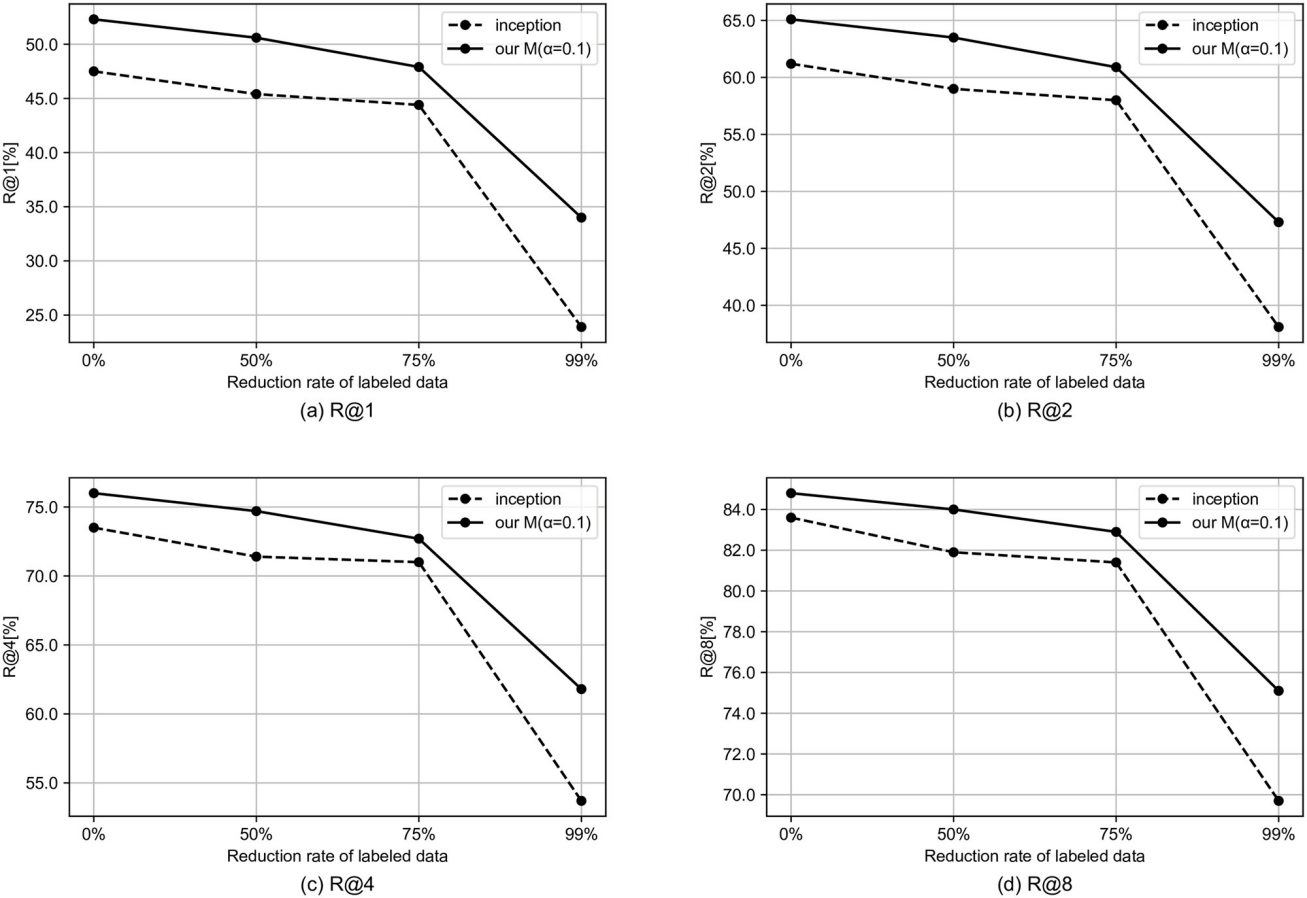
Similarity-based retrieval R@K results for semi-supervised scenario of MTG-Jamendo dataset.

**Fig 6:**
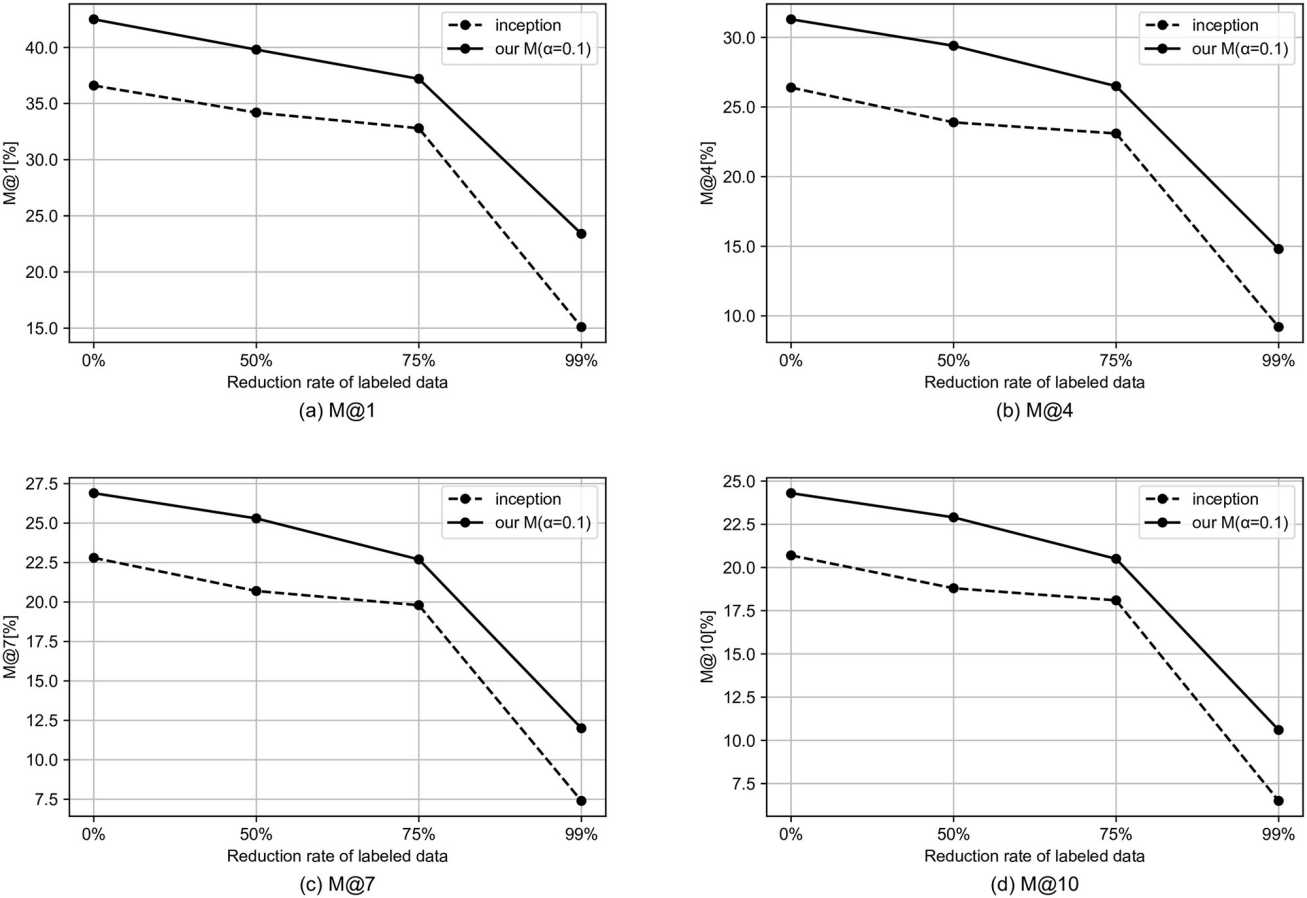
Similarity-based retrieval M@K results for semi-supervised scenario of MTG-Jamendo dataset.

**Fig 7:**
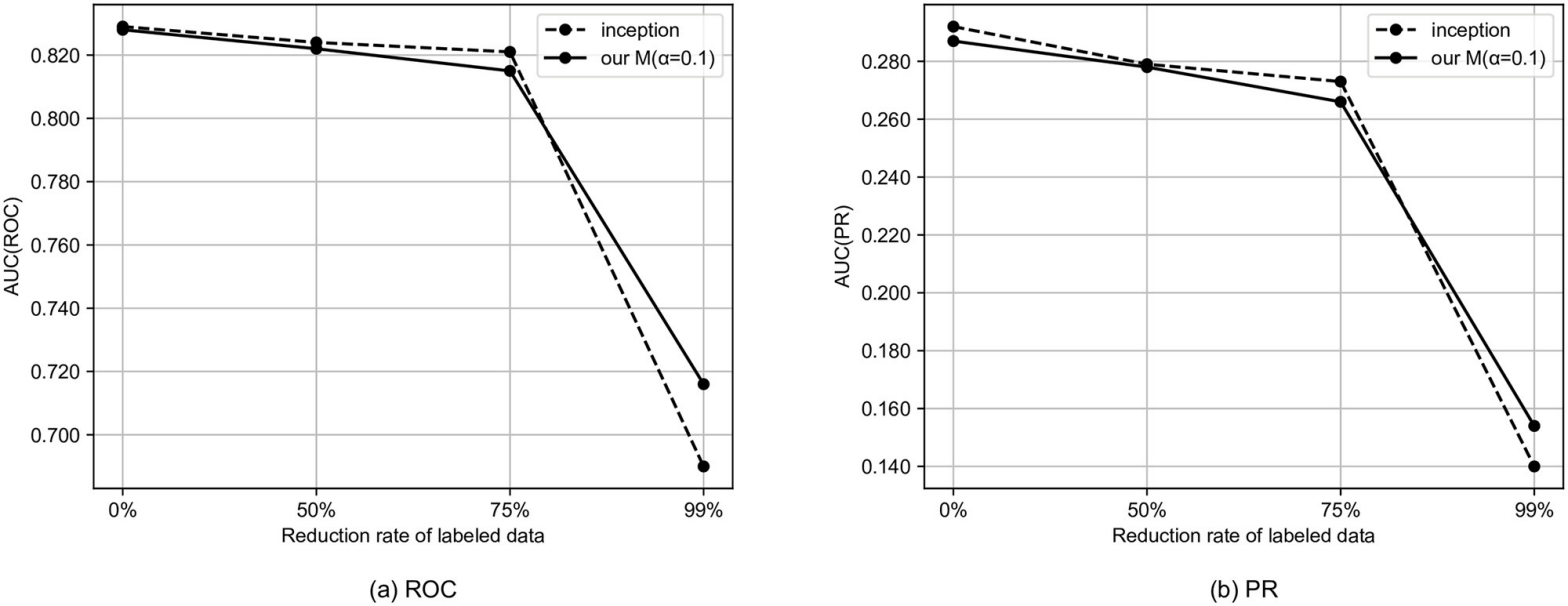
Auto-tagging results for semi-supervised scenario of MTG-Jamendo dataset.

Figs 5–7 shows the results for the semi-supervised scenario of MTG-Jamendo dataset. Similarly to the MagnaTagATune dataset, as the amount of labeled data decreases, the performance gap between our model and the baseline tends to widen, and it can be said that our method is more likely to have a larger effect when there is less labeled data.

**Fig 8:**
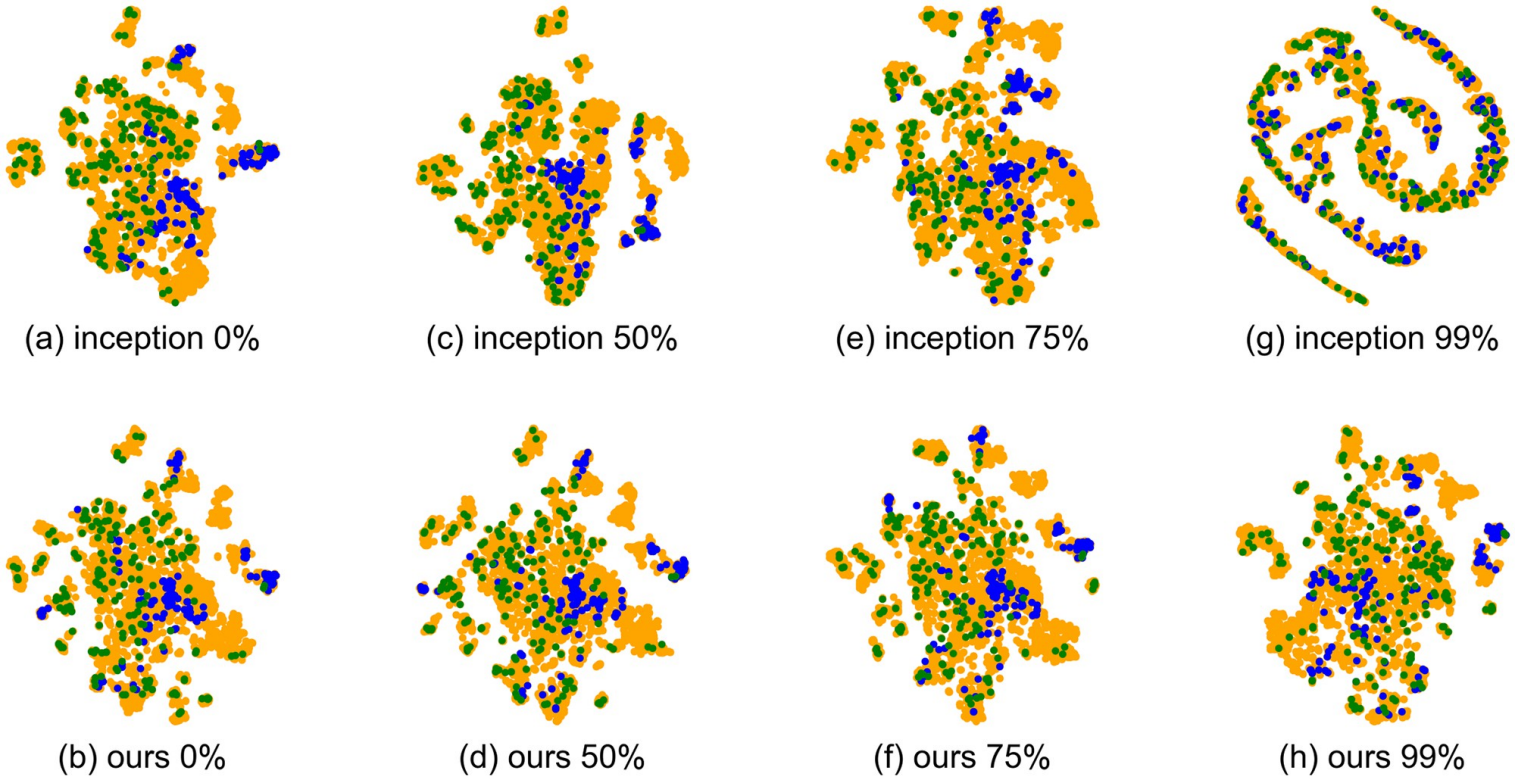
T-SNE visualization of similarity latent space Z for MagnaTagATune dataset. Green, blue, and yellow dots correspond to music tracks with ‘female vocal’ tags, ‘no vocal’ tags, and other tags, respectively. The percentage % indicates the reduction in labels used for training.

**Fig 9:**
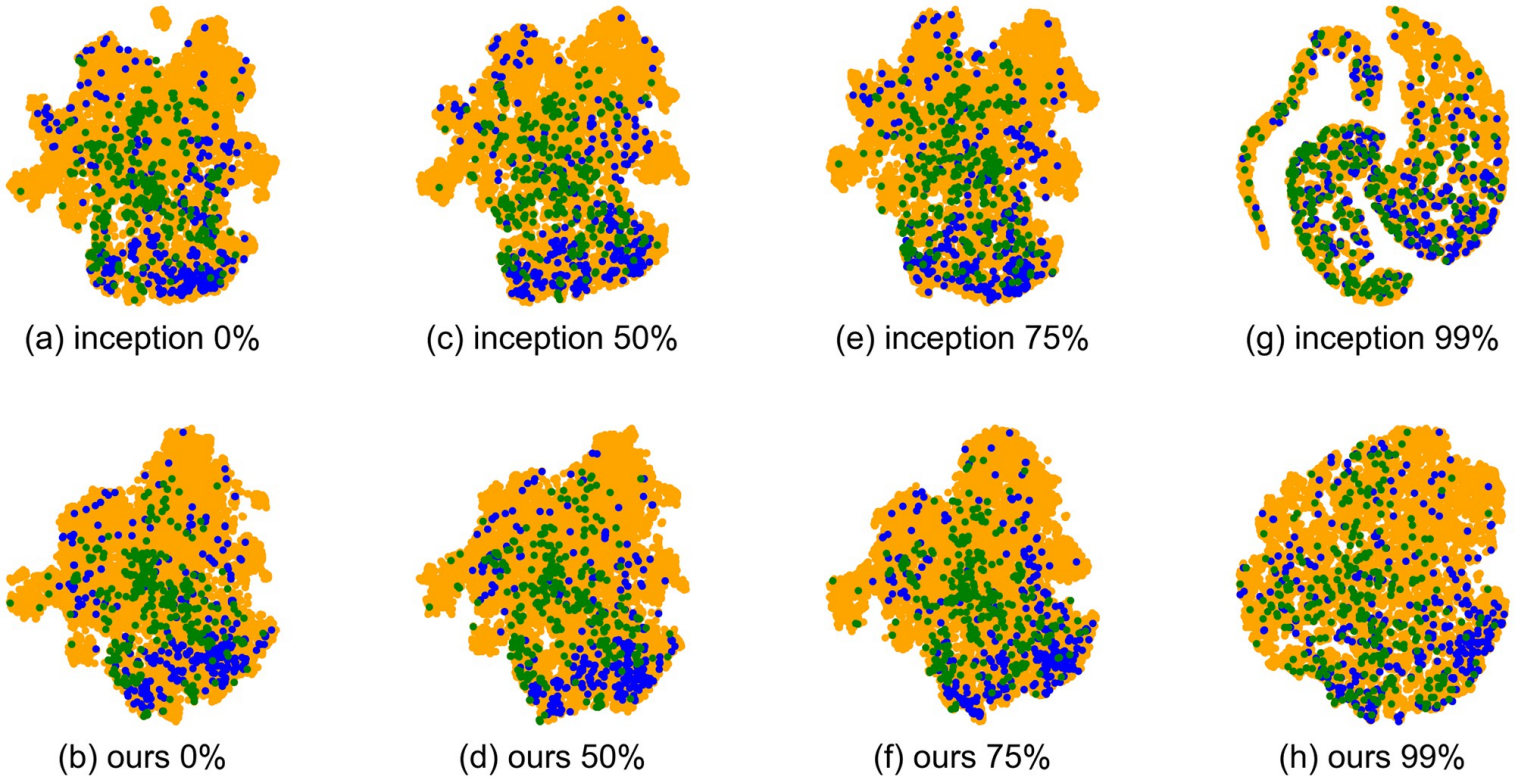
T-SNE visualization of similarity latent space Z for MTG-Jamendo dataset. Green, blue, and yellow dots correspond to music tracks with ‘instrument—voice’ tags, ‘genre—instrumentalpop’ tags, and other tags, respectively. The percentage % indicates the reduction in labels used for training.

In Figs 8 and 9, we visualize the latent space Z for similarity-based retrieval in the MagnaTagATune and MTG-Jamendo datasets, where each point in the Z space is determined by Eq (4). For visualization, we employ t-SNE, with each dot representing a music track. In the MagnaTagATune dataset (Fig 8), green, blue, and yellow dots correspond to music tracks with ‘female vocal’ tags, ‘no vocal’ tags, and other tags, respectively. In the MTG-Jamendo dataset (Fig 9), green, blue, and yellow dots represent music tracks with ‘instrument—voice’ tags, ‘genre—instrumentalpop’ tags, and other tags, respectively. We selected contrasting tags such as ‘female vocal’ versus ‘no vocal’ and ‘instrument—voice’ versus ‘genre—instrumentalpop’ for visualization because these distinctive tags are expected to be separated in the similarity latent space, providing a valuable test for evaluating the quality of the visualized latent space.

The visualization of the latent space demonstrates that when the amount of label reduction reaches 99%, the appearance of the baseline method, inception, changes significantly, while our method G or M remains relatively unchanged. Specifically, for the Inception baseline method with a 99% reduction in labels (Figs 8(g) and 9(g)), music tracks with distinctive tags such as ‘female vocal’ versus ‘no vocal’ or ‘instrument—voice’ versus ‘genre—instrumentalpop’ are mapped to less separable points, and the overall distribution of latent points of music tracks no longer appears to be tightly gathered into a single cluster.

## 7 Conclusion

In this paper, we presented a model that enhances the quality of similarity-based music retrieval and music auto-tagging. We explored the role of self-supervision in metric learning and proposed utilizing self-supervision as auxiliary loss for metric learning. Our model outperforms baseline methods and proves effective when human-provided music tags are limited. The music industry often deals with heterogeneous and extensive music databases characterized by long-tailed attributes. Human-annotated tags may be unavailable, unclean, or inconsistent across different database segments. We expect our approach, which generates learning signals without human annotation, to be effective in such real-world situations.
